# Multiple sclerosis and amyotrophic lateral sclerosis: is there an association or a red flag? A case report and literature review

**DOI:** 10.1186/s12883-024-03821-x

**Published:** 2024-08-31

**Authors:** Raseel Aljthalin, Rawan Albalawi, Atheer Alyahya, Rawabi Alhathlool, Moustafa Alhashemi

**Affiliations:** 1 Department of Adult Neurology, Prince Sultan Military Medical City, Riyadh, Saudi Arabia; 2 Faculty of medicine, University of Aleppo, Aleppo, Syria

**Keywords:** Multiple Sclerosis, Amyotrophic Lateral Sclerosis, Autonomic dysfunction

## Abstract

**Background:**

Multiple sclerosis (MS) is an inflammatory disease of the central nervous system that causes damage to the myelin and axons and is caused by genetic or environmental factors. Amyotrophic lateral sclerosis (ALS) is characterized by rapidly progressive degeneration of the motor neurons resulting in the presence of upper and lower motor-neuron signs and symptoms.

**Case presentation:**

A 46-year-old female patient presented with symmetrical weakness of the lower limbs and numbness that developed over weeks. Magnetic resonance imaging (MRI) of the brain exhibited typical demyelination features, high signal abnormality involving the periventricular and subcortical white matter, and an oval-shaped lesion. The patient was diagnosed with MS based on the clinical presentation and radiological examination. However, there was rapid progression of the symptoms, involvement of bulbar dysfunction, and muscle atrophy. Furthermore, the patient did not respond to acute therapy and immunotherapy, which made the diagnosis of MS less likely or suggested that it could be associated with another diagnosis. Her neurophysiological test met the criteria of ALS, and she was started on riluzole.

**Literature review:**

We reviewed all articles from 1986 to 2023, and there were 32 reported cases describing the co-occurrence of ALS and MS in different populations. Our case is the 33rd, and to our knowledge, it is the only case reported in the Middle East and specifically in Saudi Arabia. The main proposed mechanism according to postmortem examinations is a combination of degenerative and inflammatory processes with a cascade of production of reactive oxygen species and nitric oxide, which lead to cell death and apoptosis during concomitant ALS with MS.

**Conclusion:**

The co-occurrence of ALS and MS is extremely rare, but it can be explained by pathogenesis related to neurodegeneration, inflammation, or genetic susceptibility. Rapid progressive motor and bulbar symptoms could be red-flag symptoms, extensive evaluation might be needed for these patients.

## Background

Multiple sclerosis (MS) is an inflammatory disease of the central nervous system that causes damage to myelin and axons, influenced by genetic and environmental factors. The autoimmune process in MS leads to the degeneration of myelin sheaths. Common presenting symptoms include sensory, motor, and vision issues, as well as imbalance [1, 2].

Amyotrophic lateral sclerosis (ALS) is a neurodegenerative disease affecting the brain and spinal cord, characterized by the presence of upper and lower motor neuron signs and symptoms [3]. ALS impacts bulbar, cervical, lumbosacral, and thoracic motor neurons [4].

Both MS and ALS can exhibit non-motor symptoms. Neuropsychiatric symptoms in ALS most commonly include depression and anxiety, while cognitive impairment may arise due to frontal lobe dysfunction. Executive dysfunction is frequently reported in ALS [5]. In contrast, cognitive issues in MS can result from cortical lesions that lead to gray matter atrophy, causing a variety of cognitive symptoms. Other non-motor symptoms in both conditions include fatigue, pain, pseudobulbar affect, sialorrhea, and autonomic dysfunction [4]. Notably, autonomic dysfunction in ALS is an independent factor contributing to disease progression and is associated with more rapid rates of motor functional decline and shorter survival [6].

The co-occurrence of MS and ALS is rare; however, the proposed link involves genetic factors and demyelination activity affecting axon cells alongside the degeneration of anterior horn cells, leading to programmed cell death [3]. Activation of neuroinflammation and neurodegenerative processes due to environmental or genetic factors may explain the connection between these two diseases [7, 8]. A notable case of co-occurrence involved a patient with ALS who exhibited features of MS, potentially driven by a hexanucleotide repeat expansion of C9ORF72 [1]. This case highlighted a challenging presentation of progressive bulbar symptoms, raising questions about whether the patient was experiencing a progressive form of MS or concomitant diseases.

We report on a middle-aged woman diagnosed with MS who was later found to have ALS, along with an overview of 32 additional cases and a description of the link between these two diseases.

## Case presentation

The patient was a 46-year-old woman with a known case of type 2 diabetes mellitus. In April 2022, the patient started to have progressive symmetrical weakness of the lower limbs and numbness, which developed over weeks and was progressive in nature. She also started to have difficulty with walking and eventually required a wheelchair. She denied having any history of recent travel, raw-milk ingestion, vaccination, family history of the same presentation, illicit drug use, fever, or upper-respiratory-tract infection. She also denied having any gastrointestinal or genitourinary symptoms.

The patient was admitted to another facility, and the investigations performed included lumbar puncture and magnetic resonance imaging (MRI) of the brain. The MRI showed non-specific white-matter lesions, and the patient was diagnosed with demyelinating disease. She received pulse steroid therapy and was discharged, but there was minimal improvement regarding her muscle weakness after she went home.

**Fig. 1:**
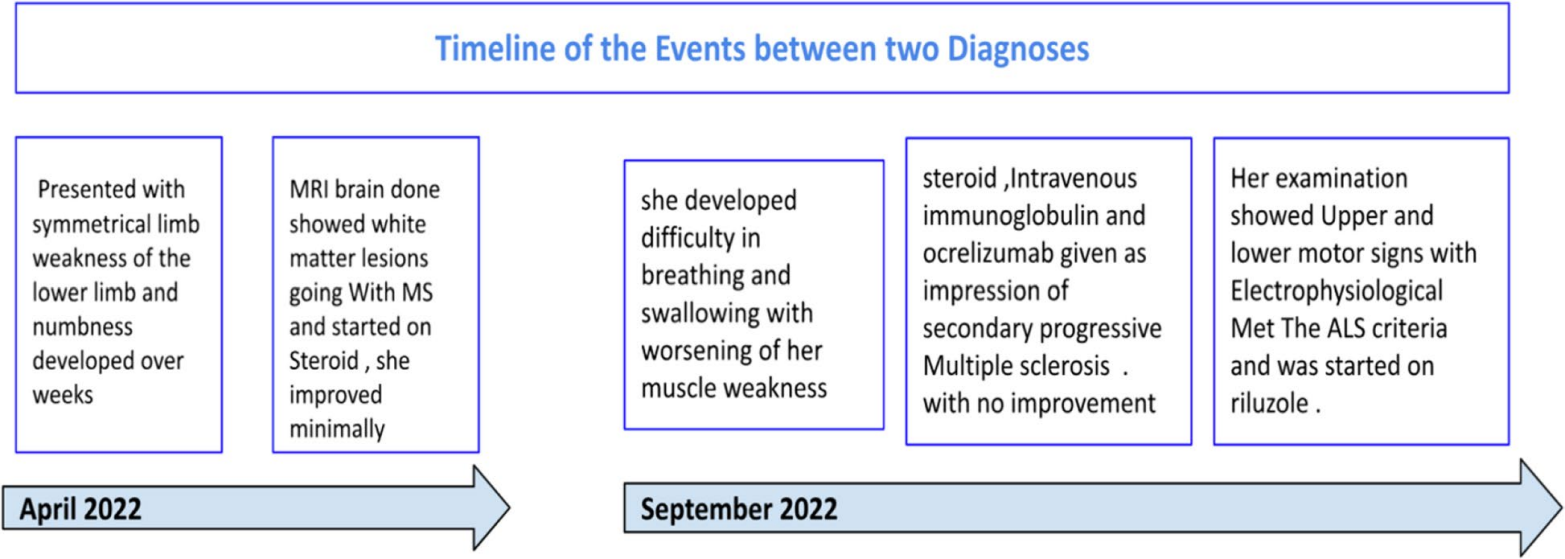
Timeline of the patient’s events

In September 2022, her weakness progressed further with upper limb weakness, and she also developed difficulty in breathing and swallowing, along with worsening of her muscle weakness. Eventually, she was tracheostomized. During her admission to the other facility, she again received pulse steroid therapy, intravenous immunoglobulin, and one dose of ocrelizumab due to an impression of secondary progressive MS. Because she was not showing any improvement in functional status, she was eventually referred to our institution for further investigations and management (Fig. 1).

### Clinical findings

The patient was on a mechanical ventilator with a trachea tube, nasogastric tube, and indwelling Foley catheter. She was awake, alert, and responding to commands. The results of cranial nerve examination were normal except for atrophy and fasciculation of the tongue with an exaggerated jaw reflex. Motor examination revealed atrophied thenar muscles, hypertonia of the upper limbs, and hypotonic lower limbs.

The Medical Research Council (MRC) grade of limb power for the upper limbs was + 2 for proximal muscles and + 3 distally at the level of the wrist and fingers. For the lower limbs, the MRC was + 2 proximally and + 1 distally at the level of the ankle, feet, and toes. The score for deep tendon reflexes was + 2 for the upper limbs with spreading reflexes, and the lower limbs were areflexic with mute plantar reflex, no clonus, and negative Hoffman sign. Sensory examination revealed decreased pinprick sensation and an absence of proprioception in the lower limb, and no sensory level was detected. The cerebellar examination was limited because of severe weakness.

### Laboratory findings

The patient’s vitamin levels were all within normal limits, including vitamin B12 and its metabolites methylmalonic acid and homocysteine. The results of a thyroid function test including thyroid antibodies were normal. Her cerebrospinal fluid (CSF) showed a normal cell count, normal levels of glucose, protein, and lactate, negative culture results, and an oligoclonal band. Moreover, the results of serological and CSF tests for *Campylobacter jejuni*, cytomegalovirus, Epstein-Barr virus, and *Haemophilus influenzae* infection were negative. Autoimmunological diagnostic including antinuclear antibody (ANA), complement fixing ANA (C-ANA), and perinuclear anti-neutrophil cytoplasmic antibodies (p-ANCA) were negative, as were tests for coeliac disease (antigliadin antibodies and antitransglutaminase antibodies). Moreover, paraneoplastic antibodies were sent through the serum and CSF, and the result was negative.

### Radiological features

**Fig. 2:**
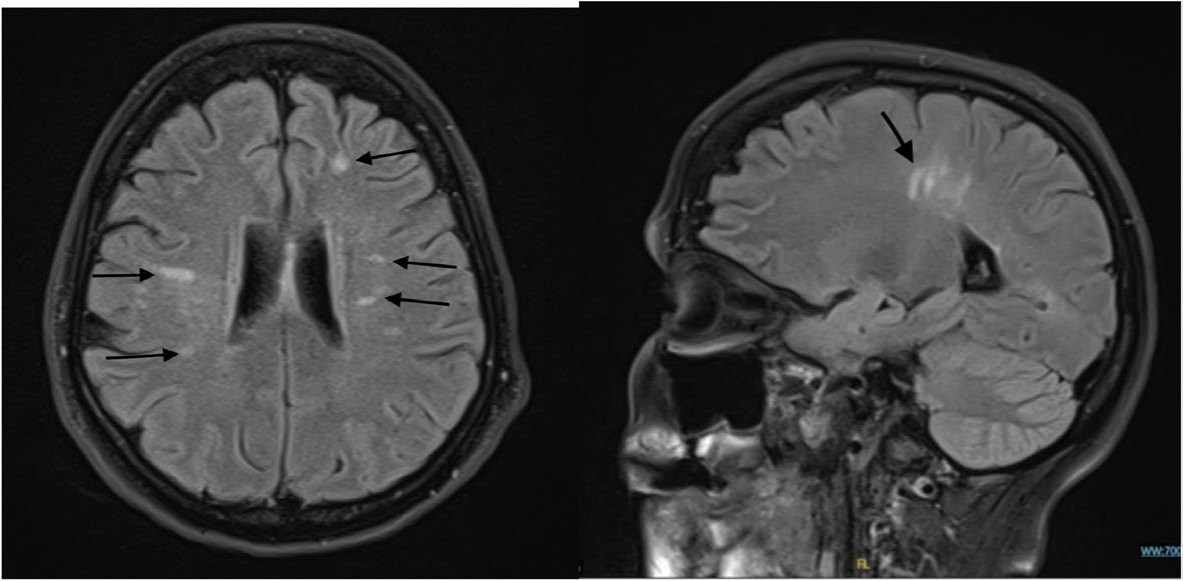
Axial and sagittal view of brain MRI showing scattered foci of hyperintense T2/FLAIR signal abnormality involving the periventricular and subcortical white matter in both cerebral hemispheres. Some of these lesions appeared perpendicular to the lateral surface of the lateral ventricle and suggested a demyelinating process

Brain and spine MRI showed evidence of scattered foci of hyperintense T2/FLAIR signal abnormality involving the periventricular and subcortical white matter in both cerebral hemispheres with limited involvement of the corpus callosum. Some of these lesions appeared perpendicular to the lateral surface of the lateral ventricle and suggested a demyelinating process. There was no pathological enhancement suggesting active disease. Imaging of the cord demonstrated normal alignment of the vertebral bodies. We performed 18-fluoro-2-deoxyglucose (FDG) whole-body positron emission tomography, which showed no detectable metabolically active lesions that could suggest malignancy in the rest of the scanned body. The MRI did not show the reason for rapid progression, and a diagnosis of MS was made based on radiological features (Fig. 2).

### Electrophysiological diagnosis

Due to the presence of both upper and lower motor signs in examination, a nerve conduction study (NCS) and electromyography (EMG) were done. The motor NCS revealed that the right median to abductor pollicis brevis was absent, the right ulnar to abductor digiti minimi showed a very small compound motor action potential of 0.4 mV, the right tibial to adductor hallucis was absent, and so was the right peroneal to extensor digitorum brevis. The sensory NCS results were normal**.**

**Fig. 3:**
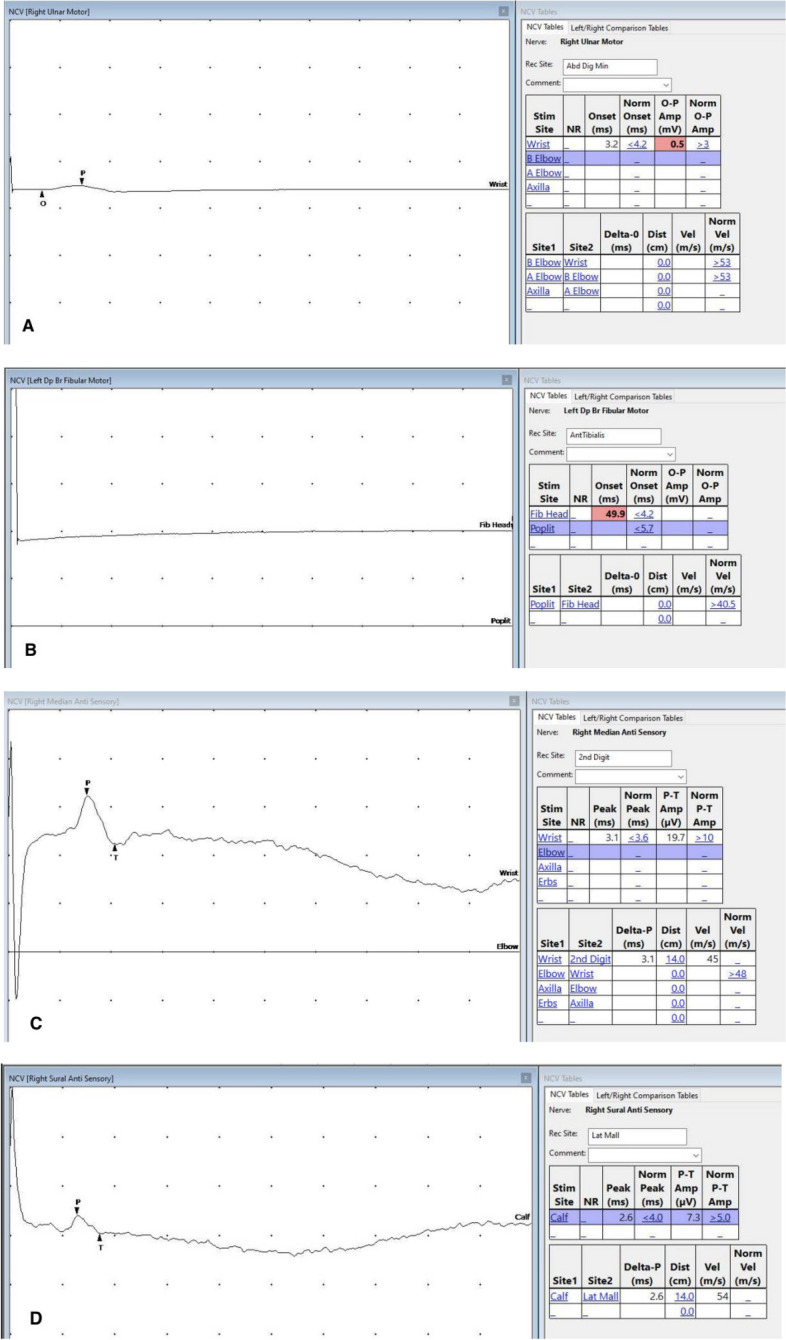
Nerve conduction study showing **A** very low compound motor action potential (CMAP) of right ulnar nerve responses, **B** absent CMAP of the left fibular nerve responses, and **C**, **D** normal sensory nerve action potential (SNAP) of upper and lower-limb sensory nerve stimulation sampled from the sural and median nerve. O: onset of the wave. P: peak of the wave. T: terminal part of the wave

In EMG, three segments were sampled. In the bulbar segment, the right tongue showed features of active denervation (+ positive sharp waves and + fibrillations). The cervical segment (right deltoid, extensor digitorum communis, and biceps) showed features of active denervation (+ positive sharp waves and + fibrillations). The lumbar segment (right tibialis anterior and vastus medialis) showed features of active denervation (+ positive sharp waves and + fibrillations). These findings are compatible with widespread motor neuron disease (Fig. 3).

## Discussion

The patient was diagnosed with MS and was managed based on the radiological features and clinical assessment. The rapid progression of the symptoms, involvement of bulbar dysfunction, and lack of response to acute therapy and immunotherapy made the diagnosis of MS less likely or suggested an association with another diagnosis. Clinical features and the results of the neurophysiological investigation met the El Escorial criteria for ALS. She was started on riluzole at 50 mg twice per day and was referred for multidisciplinary care.

The combination of MS and ALS is rare but can be explained by links between neurodegeneration, inflammation, and genetic susceptibility. In post-mortem pathological findings, demyelinating activity is observed, and degenerative processes of the anterior horn cells occur at multiple serial lumbosacral cord levels through an inflammatory cascade. This leads to the release of reactive oxygen species and nitric oxide, cell death, and apoptosis, which are observed concomitantly in ALS with MS [3–7]. In a case series study by Ismail et al., the co-occurrence of MS in a patient with ALS was explained as being driven in some way by a hexanucleotide repeat expansion of *C9ORF72.* The study highlighted that more than 1% of patients with ALS may have a history of MS [8]. Fiondella et al. reported one case of heterozygous mutation in *FUS* exon 15 [1].

So far, there have been 32 reported cases of the co-occurrence of ALS and MS in different populations, which have mainly occurred in Europe and North America. Our case is the 33rd to be reported and is the only case to our knowledge that has occurred in the Middle East and specifically in Saudi Arabia. We reviewed all related articles from 1986 to 2023. Most of the cases were females (25 out of 33), and 8 were males. The mean age of the onset of ALS is 52 years with a range of 34 to 72 years. The mean age of the onset of MS is 41 years. In some studies, no autopsies or genetic tests were performed, but clinical examinations, radiological findings, and neurophysiological observations were consistent with MS and ALS.

**Fig. 4:**
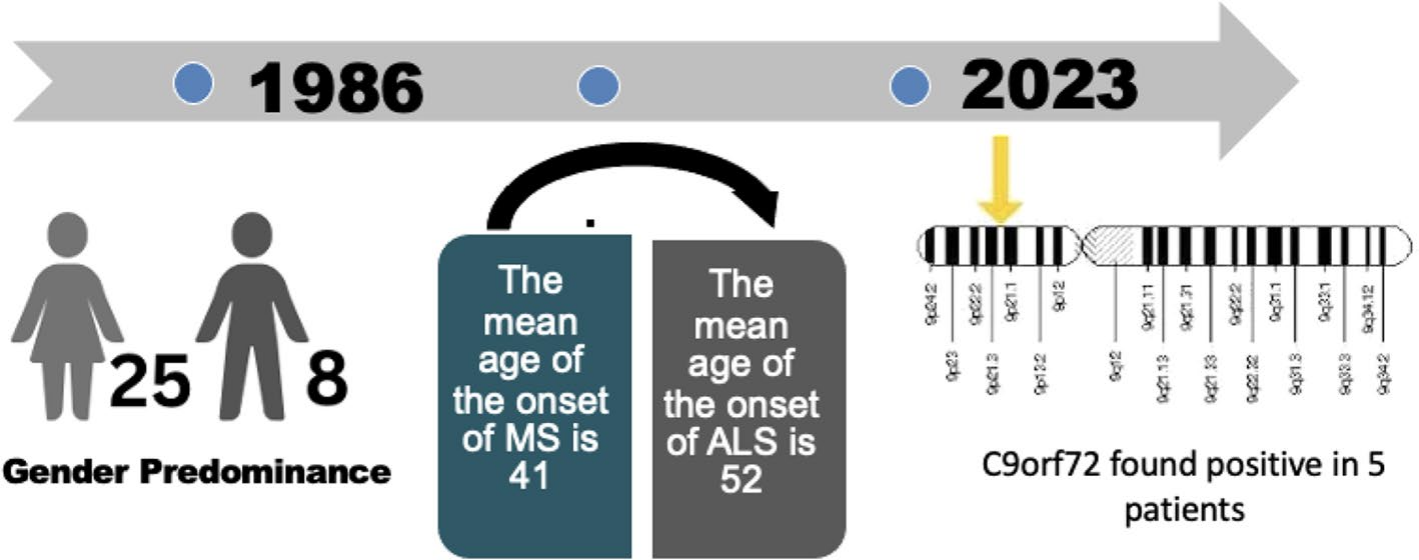
Illustration of the main characteristics of the 33 cases of MS with ALS from 1986 to 2023. There is a predominance of females (25 cases). The onset of MS occurs at an average age of 41 years, with late-onset ALS diagnosis occurring at the age of 52 years. *C9orf72* was found in only 5 cases

**Fig. 5:**
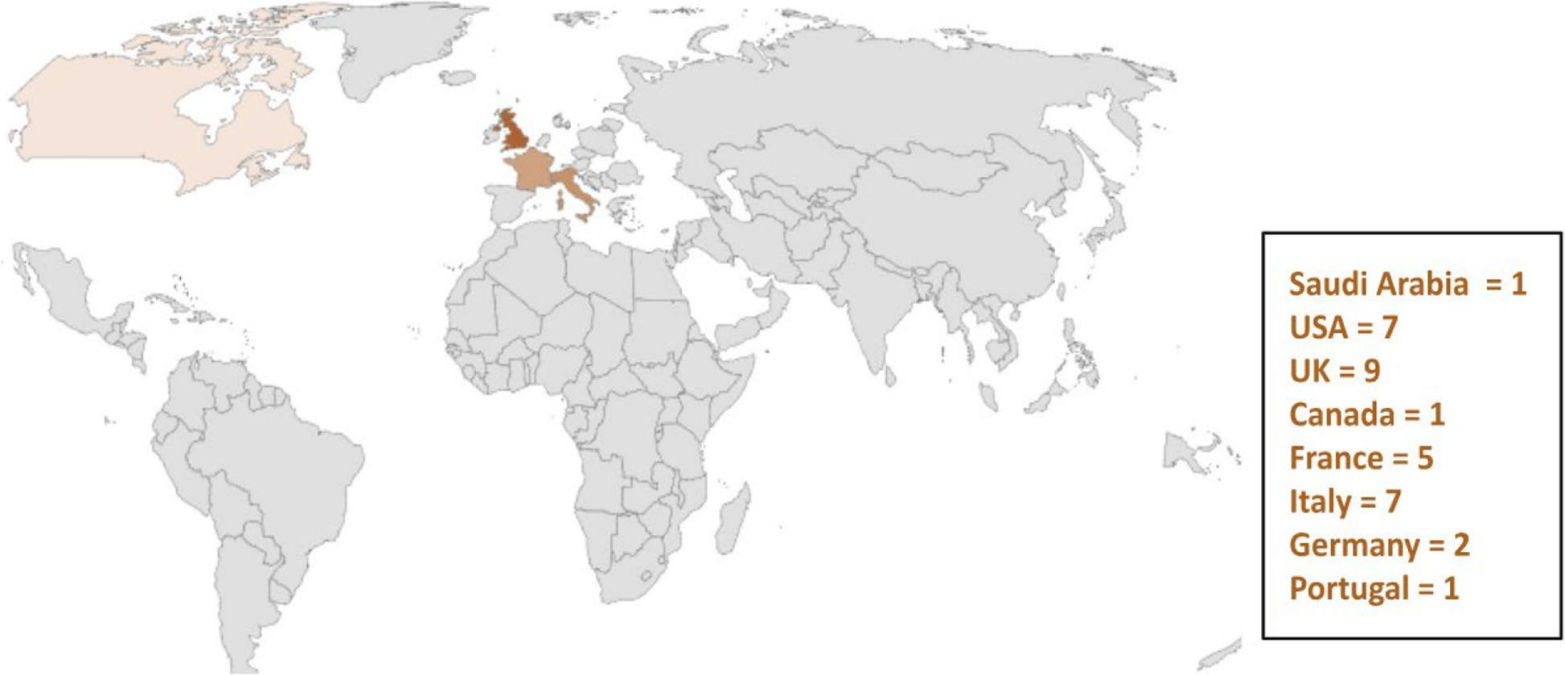
Worldwide distributions of patients with co-occurring MS/ALS diagnoses

In the different cases, patients were diagnosed with MS initially and were later diagnosed with late-onset or rapid-onset ALS. Autopsy has shown a loss of myelinated axons and neuronal loss with gliosis of the motor neuron cells [8]. Only 5 patients have been reported to be positive for *C9orf72* out of the 33 cases reviewed, but genetic tests were not done in all cases. *HLA* genotyping performed on genomic DNA has also been studied, and three cases showed positivity for HLA-B*18:01A according to Dattola et al. This antigen could play an important role in activating both neuroinflammation and neurodegenerative processes [9]. Table 1 shows the characteristics and diagnostic information of the 33 cases (Figs. 4 and 5).

**Table 1:** Demographic, clinical, and neurogenetic features of patients with co-occurring MS/ALS diagnoses

Patient	Study	Year of the study	Age	Gender	Radiographic image	CSF OCP	Electrodiagnostic	MS onset	ALS onset	Genetic test	Comment	Location
1	This study	2023	46	Female	Hyperintense T2/FLAIR signal abnormality involving the periventricular and subcortical white matter (typical demyelination)	Negative	Based on revised El Escorial classification of ALS with involvement of 3 segments	September 2022	April 2022	Not done		Saudi Arabia
2	Fiondella L et al. [1]	2022	49	Female	Demyelinating lesions in the periventricular and hemispheric deep white matter and cervical spinal cord	Positive	Presence of fasciculation and fibrillation potentials together with neurogenic signs in different districts (right deltoid, right trapezius, first interosseous and biceps bilaterally; right gastrocnemius, tibialis, and vastus medialis bilaterally)	1994 (25 y)	2017 (49 y)	Positive for *C9orf72* *mutation in FUS*		Italy
3	Hader WJ, et al. [3]	1986	56	Male	Not mentioned	Not included	EMG revealed fibrillations and fasciculations in the right deltoid and right triceps muscles; the interference pattern was reduced, and some motor units were of prolonged duration, high amplitude and polyphasic	1957 (29 y)	1982 (56 y)	Not included	Postpartum evaluation confirmed classic feature of ALS and MS Presence of demyelination plaque and loss of nerve cells in anterior horn	Canada
4	Li G, et al. [8]	2012	40	Male	Not included	Not included	Not included	2006 (37 y)	2004 (40 y)	Negative (*TDP-43* positive)	Postpartum evaluation confirmed classic feature of ALS and MS	UK
5	**Ismail A, et al.** [9]	2012	62	Female	Periventricular white matter lesions	Positive	Evidence of denervation involving cranial, cervical, thoracic, and lumbosacral segments	22	62	Positive *C9orf72*	Autopsy showed chronic plaques and severe loss of motor neurons with Bunina bodies and skein-like	UK
6	**Ismail A, et al.** [9]	2012	52	Female	Multiple lesions in the corpus callosum, cerebellum, and supratentorial lesions. High signal lesion in the right posterior aspect of cervical spinal cord (C4). No Gd-enhancing lesions	Positive	Widespread evidence of denervation was apparent on EMG	49	52	No data		UK
7	**Ismail A, et al.** [9]	2012	52	Female	Multiple periventricular and juxtacortical lesions. Multiple lesions in the cervical spinal cord and a single lesion at the dorsal spinal cord (T2)	Positive	Widespread evidence of denervation was apparent on EMG	43	52	Positive *C9orf72*		UK
8	**Ismail A, et al.** [9]	2012	67	Male	Multiple lesions in the periventricular white matter, pontine base, and cerebellar hemispheres	Positive	Widespread evidence of denervation was apparent on EMG	46	67	Positive *C9orf72*		UK
9	**Ismail A, et al.** [9]	2012	40	Male	Multiple lesions within the periventricular white matter, subcortical white matter, upper cervical cord, brainstem, cerebellum, and corpus callosum. Several of the lesions showed gadolinium enhancement indicative of active disease	Positive	Widespread evidence of denervation was apparent on EMG	39	40	No data		UK
10	**Ismail A, et al.** [9]	2012	41	Female	Multiple lesions within the periventricular white matter. No Gd-enhancing lesions	Positive	Widespread evidence of denervation was apparent on EMG	40	41	Negative		UK
11	**Ismail A, et al.** [9]	2012	56	Female	Multiple lesions within the periventricular white matter and corpus callosum. No Gd-enhancing lesions	Negative	Widespread evidence of denervation was apparent on EMG	Unknown	56	Positive *C9orf72*		UK
12	**Dattola V., et al.** [10]	2016	47	Female	Evidence of multiple demyelinating lesions	Positive	Diffuse neurogenic degeneration and signs of anterior horn cells involvement suggesting a diagnosis of ALS	48	47	*HLA B18* and* DR52* *HLA DQ6*		Italy
13	**Dattola V., et al.** [10]	2016	38	Female	MRI examination were consistent with a diagnosis of RRMS	Positive	Diffuse neurogenic degeneration and signs of anterior horn cells involvement, leading to a diagnosis of ALS	35	38	*HLA B18* and* DR52* *HLA DQ6*		Italy
14	**Dattola V., et al.** [10]	2016	52	Female	-	-	Motor axonal changes in upper and lower limbs, clearly consistent with the diagnosis of ALS	Unknown	52	*HLA DR 15*		Italy
15	**Dattola V., et al.** [10]	2016	49		MRI examination were consistent with a diagnosis of RRMS	Positive	EMG confirmed the diagnosis of ALS	46	49	*HLA DR 15* *HLA B18* and* DR52*		Italy
5	Pocock K et al. [11]	2021	72	Female	Demonstrated diffuse active and chronic denervation	Negative	Widespread active and chronic denervation	49	72	Negative for *C9orf72*	Study was included after ALS diagnosis between 2016–2019	USA
4	Pocock K et al. [11]	2021	70-	Female	Widespread active and chronic denervation	Positive	Widespread active and chronic denervation	68	70	Negative for *C9orf72*	Study was included after ALS diagnosis between 2016–2019	USA
6	Pocock K et al. [11]	2021	49	Female	Active demyelinating disease	Negative	Widespread active and chronic denervation	49	49	Negative	Study was included after ALS diagnosis between 2016–2019	USA
7	Pocock K et al. [11]	2021	51	Female	Chronic periventricular demyelinating	Not included	ALS as EMG	44	51	Negative	Study was included after ALS diagnosis between 2016–2019	USA
8	Pocock K et al. [11]	2021	64	Female	Chronic demyelinating changes in the brain and cervical spine	Not included	Widespread active and chronic denervation	63	64	Negative	Study was included after ALS diagnosis between 2016–2019	USA
10	**Sproviero W., et al.** [12]	2011	45	Female	Not included	Positive	Not included but mentioned based on clinical and electrophysiological results	Not mentioned	45	*P525L* mutation (*ALS-04*)		Italy
11	**Guennoc A. M, et al.** [13]	2018	53	Female	T2-weighted periventricular and left parietal lobe white matter hyper-signals	Not included	chronic denervation at the bulbar, cervical, and lumbar levels	2003 (41 y)	2014 (52 y)	-		France
12	**Guennoc A. M, et al.** [13]	2018	52	Male	MRI findings revealed characteristic periventricular hyper-signals in T2-weighted sequences	-	Widespread denervation without conduction abnormalities	34	50	-		France
13	**Guennoc A. M, et al.** [13]	2018	51	Female	Active demyelination at cervical region with stable cortical lesion	-	-	39	51	-		France
14	**Guennoc A. M, et al.** [13]	2018	60	Female	Chronic demyelinating changes in the brain (periventricular)	-	-	27	59	-		France
15	**Guennoc A. M, et al.** [13]	2018	54	Female	Periventricular and pericallosal hypersignals in T2- weighted sequences with some lesions enhanced after gadolinium injection	Negative	Diffuse denervation result supported the diagnosis of ALS	54	55	-		France
16	**Hewitt C, et al. ** [14]	2010	62	Female	Magnetic resonance imaging of the brain and spine at presentation demonstrated demyelination consistent with the earlier diagnosis of multiple sclerosis	-		23	62	*Gly174del*	Pathologic evidence of both ALS and multiple sclerosis. Loss of LMNs from the spinal cord and medullary motor nuclei was associated with Bunina bodies and ubiquitin/TDP-43–positive	UK
17	**Allen J. A., et al.** [15]	2007	51	Male	Periventricular changes with involvement of cervical spine lesions	Positive	Chronic and active denervation is present in three limbs, with profuse fibrillation potentials within paraspinal musculature. Spontaneous activity	27 y	2004 (51 y)	-		USA
29	**Trojsi F., et al.** [16]	2012	34	Female	Lesions on periventricular or juxtacortical white matter and gadolinium enhancement of the lesion in the right corona radiata	Positive	Pathological spontaneous activity at rest (fibrillations and fasciculations) and chronic, neurogenic motor unit changes in three sites (bulbar, upper, and lower limbs),	33	34	Negative		Italy
30	**Dynes G. J et al.** [17]	2000	62	Female	Lesions in the callosal and pericallosal regions with two enhancing periventricular foci. also, multiple lesions in cervical and thoracic spine	Positive	Diffuse fasciculations with fibrillations, reduced recruitment and chronic, neurogenic motor unit changes in both arms and the left leg	61	61	No data	Autopsy highlighted loss of myelinated axons in the lateral and anterior corticospinal tracts with Marked neuronal loss and gliosis were seen in the anterior horns	USA
31	**Machner B., et al.** [18]	2007	56	Female	Multiple periventricular white matter and cervical lesions	Positive	Chronic signs of denervation in all limbs without nerve conduction block. Clinical and paraclinical examination met the El Escorial criteria for ALS	55	56	-		Germany
32	**Borisow N., et al.** [19]	2013	56	Male	T2-hyperintense lesions located juxtacortically, periventricular, and in the area of the optic radiation	Positive	Spontaneous activity in upper and lower limb	Unknown	2011 (55 y)	-		Germany
33	**M. Soares, M, et al.** [20]	2022	52	Female	Hyperintense lesions predominantly affecting the periventricular white matter (Dawson's fingers) and brainstem	-	Marked loss of motor units with signs of reinnervation in tongue, chronic neurogenic motor units potentials with fasciculation potentials and signs of acute denervation (fibrillation and sharp-waves) in proximal and distal muscles of upper and lower limbs, bilaterally	1999	2021 (52y)	*C9orf72* expansion was negative,	Positive spastic paraplegia 11 (SPG11),	Portugal

## Conclusion

The co-occurrence of ALS and MS is extremely rare, but it can be explained by a mix of pathogenesis involving neurodegeneration, inflammation, and genetic susceptibility. Careful evaluation is needed for patients with rapid progressive motor and bulbar symptoms who are initially diagnosed with MS as this presentation could be a red flag. Extensive evaluation might be needed for these patients. To gain more understanding of the co-occurrence, pathological testing, genetic testing, and *HLA* genotyping should be considered for diagnosis.

## Data Availability

All data generated or analysed during this study are included in this published article and its supplementary information files.

## Data availability

No datasets were generated or analysed during the current study.
